# Reliable detection of CNS lymphoma-derived circulating tumor DNA in cerebrospinal fluid using multi-biomarker NGS profiling: insights from a real-world study

**DOI:** 10.1186/s40364-025-00777-z

**Published:** 2025-05-09

**Authors:** Veronika Navrkalova, Andrea Mareckova, Samuel Hricko, Viera Hrabcakova, Lenka Radova, Vaclav Kubes, Jakub Porc, Tomas Reigl, Sarka Pospisilova, Jana Kotaskova, Andrea Janikova

**Affiliations:** 1 Department of Internal Medicine– Hematology and Oncology, University Hospital Brno and Faculty of Medicine, Masaryk University, Brno, Czech Republic; 2 Center of Molecular Medicine, CEITEC - Central European Institute of Technology, Masaryk University, Brno, Czech Republic; 3 Department of Medical Genetics and Genomics, Faculty of Medicine, Masaryk University and University Hospital Brno, Brno, Czech Republic; 4 Department of Pathology, University Hospital Brno, Brno, Czech Republic

**Keywords:** ctDNA detection, Liquid biopsy, Multi-target NGS, Clinical practice, CNS lymphomas

## Abstract

**Background:**

Diagnosing primary or secondary CNS lymphoma (CNSL) is a clinical challenge due to the limitations of standard biopsy and imaging procedures despite established guidelines. Therefore, accurate biomarkers and analytical methods that are convenient for practical routine use are needed to diagnose and manage these aggressive lymphomas effectively. We evaluated the utility of minimally invasive circulating tumor DNA (ctDNA) detection in a prospective real-world scenario, moving this approach closer to clinical practice.

**Methods:**

A total of 164 plasma, cerebrospinal fluid (CSF), and tumor samples from 56 CNSL patients were collected to analyze tumor DNA by the diagnostic next-generation sequencing (NGS) panel LYNX, enabling simultaneous analysis of gene variants, chromosomal aberrations, and antigen receptor rearrangements in targeted regions.

**Results:**

The well-known genetic heterogeneity of CNSL was refined with integrative molecular data, showing the most frequent *MYD88*, *PIM1*, and *KMT2D* mutations and a broad spectrum of chromosomal aberrations, reflecting high genomic complexity. The multi-target approach achieved a substantially higher detection rate of CNS infiltration (90%) than tracking a single variant in gene *MYD88* (46%). CSF clearly surpasses plasma if applying a routine (non-ultrasensitive) NGS approach and allows for more reliable evidence of CNS involvement than conventional flow cytometry (91% vs. 21%, *p* < 0.001). Parallel analysis of tumor DNA in both cell-free and cellular DNA from CSF makes the probability of primary or secondary CNS malignancy detection even higher.

**Conclusions:**

Our prospective, tissue-agnostic approach highlights the feasibility of ctDNA sequencing by a commonplace and affordable method, offering higher sensitivity to detect CNS infiltration with lymphoma than standard cell-analyzing techniques. We accentuate the benefit of a multi-target NGS approach and adequate CSF sampling to obtain satisfactory diagnostic yield. Less invasive liquid biopsy testing by comprehensive NGS complements standard procedures in the diagnostics and management of CNSL patients, especially when encountering limitations.

**Supplementary Information:**

The online version contains supplementary material available at 10.1186/s40364-025-00777-z.

## Background

The central nervous system (CNS) invasion with B-cell lymphoma is a rare but clinically challenging scenario with poor patient outcomes [1]. Depending on the original localization of the tumor, it can manifest as a primary CNS lymphoma (PCNSL) restricted to CNS without systemic disease or as a secondary CNSL lymphoma (SCNSL), representing synchronous CNS and systemic involvement or more frequent isolated CNS relapse [2]. CNSLs are mostly of DLBCL (diffuse large B-cell lymphoma) histology and account for 6–7% of all brain tumors [3]. The historically dismal prognosis of CNS lymphomas has improved with intensified treatment regimens, with current median survival reaching approximately 3–4 years in PCNSL patients and 2–3 months in cases of CNS recurrence in systemic lymphoma [4–6]. To cure these aggressive lymphomas of CNS is difficult, with fewer options than for systemic DLBCL; however, new possibilities of targeted therapy based on underlying biology [7] may change the prognosis in the future.

Traditional methods of diagnosing and monitoring CNSL, such as excisional biopsy, magnetic resonance imaging (MRI), and cerebrospinal fluid (CSF) cell analysis, have specific limitations [8, 9]. The neurosurgical procedure is not without risks, can lead to insufficient material quantity or could be unfeasible due to anatomically inaccessible locations. MRI shows insufficient discrimination ability and, along with standard evaluation of CSF by cytology or flow cytometry (FC), also limited sensitivity, which is evident from disease recurrence after negative findings with those methods [10]. Corticosteroid therapy can additionally cause diagnostic delay as it blurs neuroimaging and impacts tissue morphology [11]. Consequently, there is a growing interest in minimally invasive techniques of cell-free DNA (cfDNA) analysis from liquid biopsies to reliably identify brain lymphoma in clinical settings [12–14].

Next-generation sequencing (NGS) of circulating tumor DNA (ctDNA), representing a fraction of the overall cfDNA pool, has recently been shown as a promising biomarker in solid and hematologic tumors, with most evidence from systemic DLBCL [15–17]. In brain tumors and metastasis, there is growing interest in the potential utility of ctDNA profiling from plasma and CSF and its contribution to accurate diagnosis and management [12–14, 18, 19]. However, particularly in CNSL, a debate concerning appropriate body fluid and the sensitivity of used methods to reach affordable and practical approach is still ongoing. Previous studies are limited by low numbers of paired CSF-plasma samples, single-target assays, suboptimal ctDNA detection levels, or the need for demanding ultrasensitive methods [12–14, 20–23].

PCNSL and SCNSL are characterized by the overlapping portfolio of recurrent aberrations that resemble DLBCL molecular subtype MCD defined from tissue in the study by Wright et al. [24]. Mutations in genes *MYD88*,* CD79B*,* PIM1*, 9p deletion (*CDKN2A* locus), 6p deletions (*HLA* locus), etc., correspond with activated BCR signaling, NFκB pathway, and immune evasion despite the localization in the immune-privileged site [7]. *MYD88* L265P variant represents a specific diagnostic marker of CNSL but has suboptimal sensitivity in ctDNA [25–27]. Therefore, multiple target assays [28] or ultrasensitive methods [13] are being developed to overcome this limitation and enhance the detection rate.

Taken together, current CNSL studies demonstrate that ctDNA can be detected in both plasma and CSF, though with methods of various sensitivity, and could be used to monitor the disease and predict patient outcomes. Our aim was to assess the relevance of ctDNA analysis in CSF and plasma in real-world CNS lymphoma diagnostics using a routine NGS tool that covers a broad spectrum of lymphoma biomarkers. We conducted tissue-agnostic comprehensive molecular profiling of liquid biopsies and explored the benefits over standard circulating tumor cell (CTC) analysis and imaging techniques. We also focused on practical laboratory aspects of liquid biopsy sampling and testing from a routine perspective. Finally, we compared our results among PCNSL and SCNSL patients with diagnosed or suspected CNS involvement to assess clinical utility and profit in managing those patients.

## Materials and methods

### Patients and material

At the time of initial diagnosis or CNS relapse, we collected 164 parallel samples from 56 CNSL patients, including 61 CSF, 61 plasma, and 42 formalin-fixed paraffin-embedded (FFPE) blocks. Patients were consecutively diagnosed and treated at the Internal Hematology and Oncology Department, University Hospital Brno (Czech Republic), during the years 2021–2023. All individuals signed a written informed consent in accordance with the Declaration of Helsinki, and the study was approved by the local ethics committee (23-090621-EK). Here, we present a real-world scenario where patients underwent routine diagnostic procedures, examinations, and management according to the local consensus guidelines of the Czech Lymphoma Study Group.

Twenty-five patients were diagnosed with PCNSL and 31 had systemic B-cell lymphoma (mostly DLBCL) with proven or suspected CNS involvement (hereinafter referred to as “SCNSL subgroup”), including 16 patients analyzed within a relapse. Three patients had available serial samples from both diagnosis and relapse (Table 1). All patients underwent standard staging procedures based on MRI of the brain and chest-abdomen-pelvis CT; in some patients, whole-body PET/CT or PET/MRI was performed. Lumbar puncture was performed as a standard examination in case of PCNSL diagnosis and high risk or suspicion of CNS involvement in SCNSL (clinical symptoms, CNS-IPI 4-6, testicular or spinal involvement) before immunochemotherapy initiation. Twenty-eight patients received corticosteroid treatment before CSF collection (8 mg dexamethasone 3x/day, median duration 5 days). Liquid biopsies were collected prospectively, while FFPE blocks were retrospectively taken from the biobank. The median age at the time of sampling was 67 years (range 18–79), 57% were male, and the median follow-up of the entire cohort was 14 months (Table 1; further details on patients and sampling are provided in Suppl. table S1).

**Table 1:** Overview of basic clinical and laboratory parameters of CNSL patient cohort (*n* = 56) (detailed data in table S1)

Sex: male / female	32 / 24
**Age**: median (range)	
at diagnosis	65 (18–79) years
at sampling	67 (18–79) years
**Time initial dg - sampling: median** (range)	
PCNSL	0 (0–64) months
SCNSL	3 (0–91) months
**Initial lymphoma diagnosis**:	
PCNSL	25
SCNSL	31
- DLBCL	19
- BL	3
- HGBL	2
- others	7
**Newly diagnosed / relapsed**	40* / 16
PCNSL (*n* = 25)	22 / 3
SCNSL (*n* = 31)	18 / 13
**Follow-up**: median (range)	14 (2-109) months

* three patients had serial samples from diagnosis and relapse (1 PCNSL, 2 SCNSL)

### Sample processing, cfDNA isolation, and DNA extraction

**Fig. 1:**
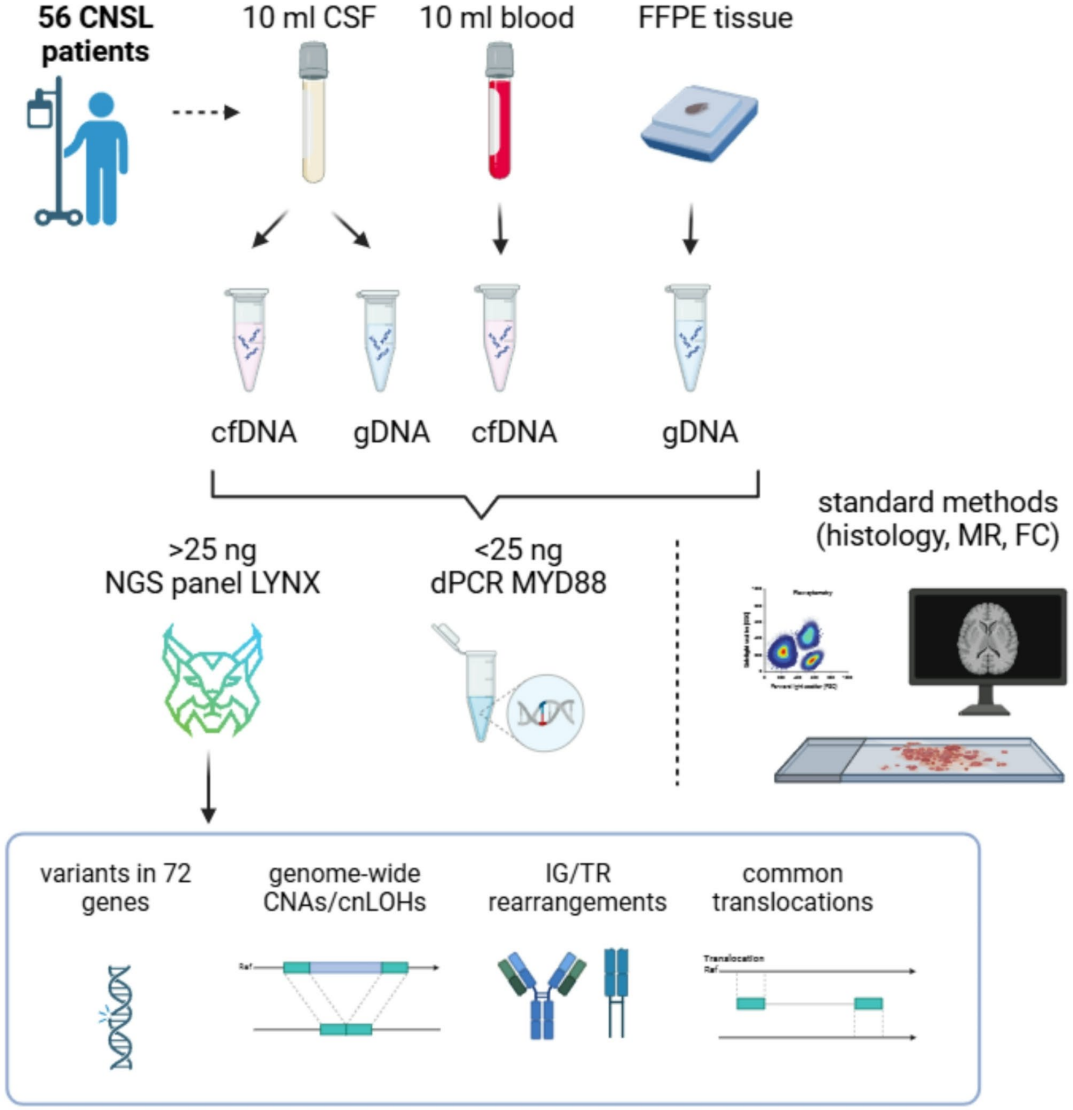
Workflow of sample processing and analytical methods used to detect ctDNA in CNSL patients

Liquid biopsy samples were collected into special tubes, which preserve cfDNA and prevent genomic DNA release (STRECK, Nebraska, US). Peripheral blood was centrifuged twice (300 g for 20 min, followed by 5000 g for 10 min at room temperature) to obtain plasma samples. The median volume of CSF and plasma was 10 ml (range 2–11) and 4 ml (range 1–8), respectively. CSF samples were centrifuged twice in the same conditions as blood to separate the supernatant for cfDNA extraction and sediment for genomic DNA (gDNA) extraction. CSF and plasma-derived cfDNA were isolated using a QIAamp Circulating Nucleic Acids kit (Qiagen, Hilden, Germany), quality-checked on cfDNA ScreenTape assay (Agilent, Santa Clara, CA), and quantified using a Qubit fluorimeter (Thermo Fisher Scientific Inc., Waltham, MA). gDNA from CSF sediment and FFPE blocks were extracted using the QIAamp DNA Blood Mini Kit and the QIAamp DNA FFPE tissue kit (both Qiagen), respectively. The schematic study workflow is shown in Fig. 1.

### Comprehensive NGS profiling and digital PCR (dPCR) for ctDNA detection

To identify ctDNA presence in the cfDNA from collected samples, we screened for recurrent aberrations by the customized capture-based NGS panel LYNX [29] or used dPCR for the *MYD88* L265P variant when an insufficient amount of cfDNA was isolated (approx. <25 ng) (Fig. 1). NGS library preparation and data analysis was performed as previously described [29] with updated and validated design of target regions (1.13 Mb in total) (Suppl. Figure 1). The LYNX panel is a routine research and diagnostics tool employed at our clinic, enabling integrative analysis of gene variants (LOD 5%), chromosomal alterations (copy number alterations, CNAs; copy-neutral loss of heterozygosity, cnLOHs; LOD 15–20%), translocations (LOD 5%), and antigen receptor rearrangements that are typical for lymphoproliferative disorders. This broad spectrum of targets enhances the sensitivity of ctDNA detection above the validated detection limit of individual variants. Throughout the manuscript, the positive ctDNA infiltration of CSF or plasma was concluded if at least one of the ctDNA markers was identified above the detection limit of a method. Levels of ctDNA were expressed in haploid genome equivalents per milliliter (hGE/ml) of CSF or plasma, calculated from cfDNA concentration and the mean allele fraction of somatic reporters [17].

### Flow cytometry of CSF samples

All CSF samples screened for the ctDNA presence were analyzed by an 8-color FC panel. The presence of lymphoma cells in CSF was tested using a combination of the following antibodies: CD3, CD10, CD19, CD20, CD235 (GlyA), CD45, sKappa, and sLambda chain. All antibodies were used in concentrations recommended by the manufacturer. After the incubation, the cells were washed using a phosphate-buffered saline solution (pH 7,4). FC data was acquired using a BD FACSCanto II flow cytometer (BD Biosciences, USA). The maximum of present leucocytes (up to 50,000) was analyzed in each sample. Data was evaluated with BD FACSDiva™ (BD Biosciences).

### Statistical analysis

Subgroups of categorical variables were compared using Fisher’s exact and continuous variables using Mann-Whitney U tests. The correlation between continuous variables was evaluated by the Spearman test. The log-rank test was applied for survival difference comparisons, and survival curves were visualized using the Kaplan-Meier method. Overall survival (OS) was calculated from diagnosis to death from any cause, while event-free survival (EFS) was defined only for SCNSL patients as the time from diagnosis to CNS relapse or lymphoma-associated death. Both OS and EFS were censored at the time of the last appointment. Statistical analyses were performed using free software R [30]. The *p*-value < 0.05 was considered statistically significant. Schematic figures were created using Biorender (BioRender.com).

## Results

### The amount of cfDNA and ctDNA in CSF is equal in PCNSL and SCNSL and differs from plasma level

CSF and plasma were collected simultaneously during patient enrollment into the study. In general, the total cfDNA amount in CSF per sample (*n* = 61, median 109 ng, range 6-2900) was notably lower than in plasma samples (*n* = 61, median 571 ng, range 21-10550, *p* < 0.001). When the PCNSL and SCNSL groups were compared, no difference was observed in CSF (median 19.7 ng and 19.3 ng, respectively; *p* = 0.99), in contrast with an expected trend towards a higher level in plasma of SCNSL patients (median 93.5 ng and 140.0 ng, respectively; *p* = 0.08) (Suppl. Figure 2A and 2B).

Out of 61 CSF samples, 31 were eligible for NGS analysis (> 25 ng of cfDNA), 11 to dPCR only, and 19 (31%) could not be analyzed due to scarcity of cfDNA. Almost all plasma-derived cfDNA samples (60/61) were analyzed by NGS. The median of ctDNA fraction detected in CSF was 4.6 log hGE/ml (range 3.4–6.6, *n* = 29) and 5.8 log hGE/ml (range 4.1–7.4, *n* = 18) in plasma. ctDNA did not differ among PCNSL and SCNSL in CSF (median 4.5 vs. 4.6 log hGE/ml, *p* = 0.68) (Suppl. Figure 2 C). In plasma, ctDNA was detected only among SCNSL cases and in one PCNSL patient.

A positive correlation was observed between ctDNA and cfDNA levels in both CSF and plasma (*p* < 0.003 and *p* < 0.001, respectively). Corticosteroid treatment, which was applied in 74% (28/38) of patients with CNS involvement evidenced by standard methods, did not influence the levels of cfDNA (*p* < 0.14) and ctDNA (*p* < 0.98) in CSF samples.

### ctDNA detection by tumor-agnostic approach accentuated CSF relevance over plasma in CNSL

Without prior knowledge of tumor-associated markers from tissue biopsy, the LYNX panel and dPCR revealed the presence of ctDNA in the majority of eligible CSF samples with reliable output (29/32, 91%) (Suppl Table S2). gDNA from CSF sediments, if obtainable, was also analyzed by NGS (45 out of 61 samples), detecting tumor-specific alterations in 42% (18/43) cases with good-quality NGS data.

**Fig. 2:**
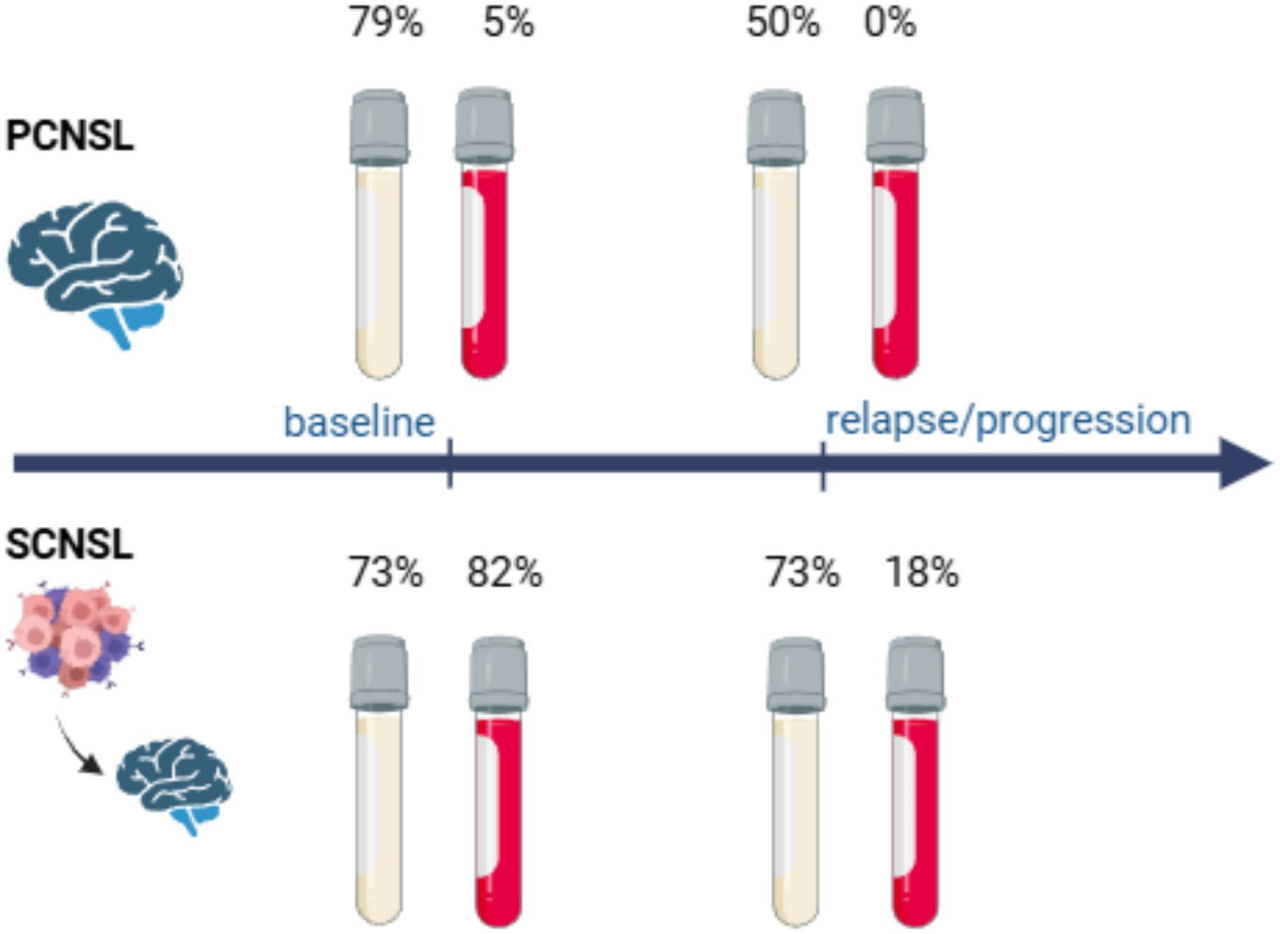
Tumor DNA detection rate in liquid biopsies according to the primary or secondary CNS involvement and sampling during the disease course. The CSF sample is a yellow tube, and the blood sample is a red tube (all tubes are special tubes for cfDNA preservation). In CSF, tumor DNA was detected in cfDNA and/or gDNA (details are dissected in the Supplementary Table S2)

We then dissected our results according to the type of CNS involvement and timing of sample collection during the disease course (Fig. 2, Suppl Table S2). At the baseline, PCNSL patients showed tumor infiltration predominantly in CSF (79%, 15/19; jointly from cfDNA and/or gDNA) and only very rarely in plasma (5%, 1/22). Baseline SCNSL samples were comparably positive in CSF (73%, 8/11) and significantly more positive than PCNSL cases in plasma (82%, 14/17). At the time of progression/relapse of systemic lymphoma in the brain, ctDNA was detected mainly in CSF (73%, 11/15) but rarely in plasma (18%, 3/17). Remarkably, among the 14 SCNSL cases with detectable CSF-ctDNA, seven showed no evidence of CNS involvement by standard methods. Conversely, one patient with MRI-confirmed CNS involvement had undetectable ctDNA (Suppl. Table S3).

It is worth mentioning that the analysis of cellular gDNA from CSF pellet can help detect tumor presence but is not as reliable as ctDNA from the supernatant fraction. Firstly, cells do not have to be present in sufficient amounts, and secondly, tumor cells can be sparse in the overall pool. We analyzed 26 patients with paired material available, and 12/26 (46%) cases showed discordant results, where positive tumor infiltration was detected only in ctDNA but not in gDNA (Suppl Table S2).

### CSF-derived cfDNA analysis is more sensitive than flow cytometry to discern CNS infiltration

**Fig. 3:**
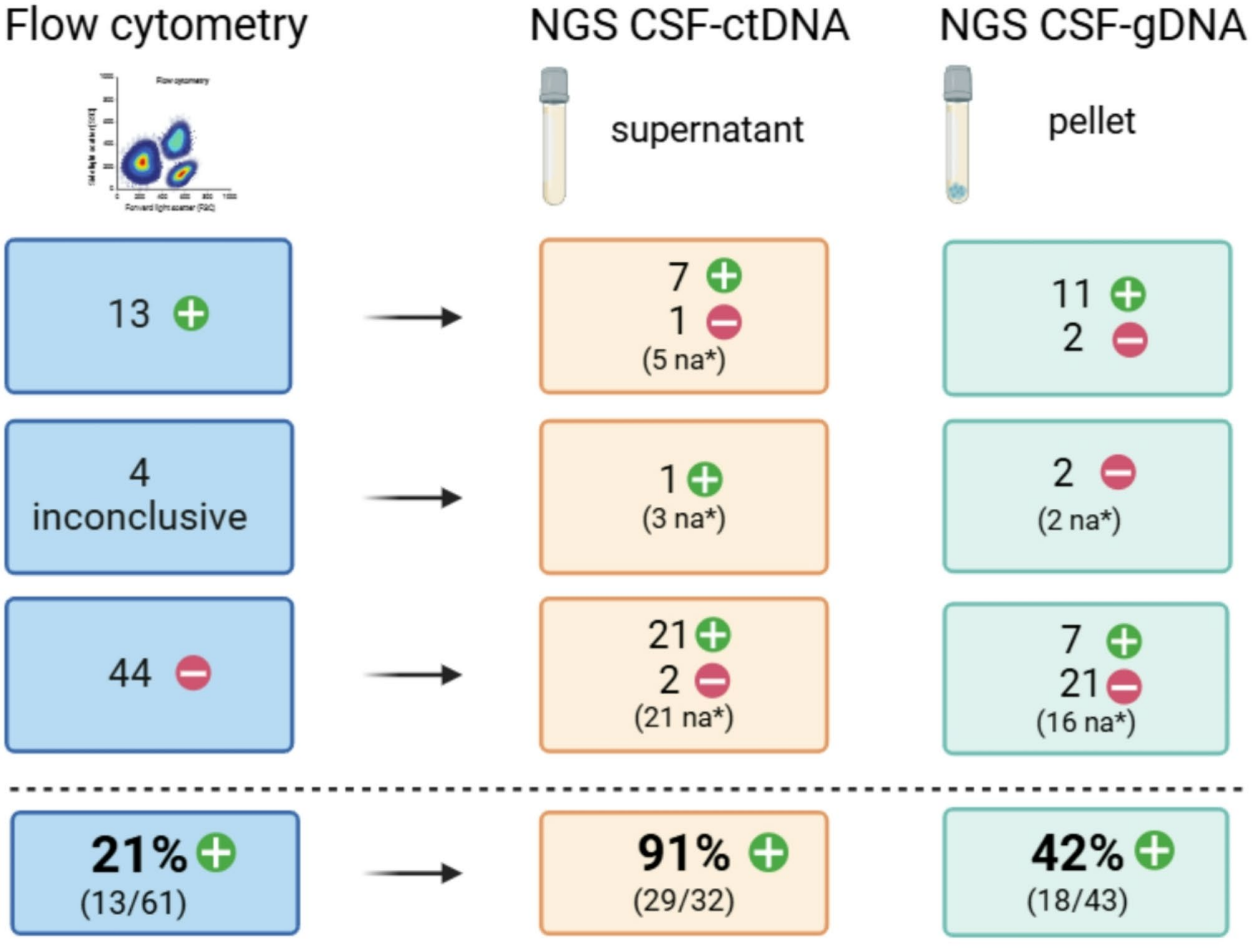
Sensitive detection of tumor infiltration by comprehensive NGS analysis of CSF ctDNA compared to flow cytometry. na* not analyzed due to the low amount of cfDNA/gDNA for NGS

To assess the clinical utility of ctDNA NGS analysis in a routine setting, we compared the number of positive cases detected from liquid biopsies to flow cytometry results. Out of 61 CSF samples, FC identified lymphoma cells in 13 patients (21%), 44 (72%) were negative, and 4 had inconclusive findings. Cell-free DNA NGS revealed more positive cases with a significant increase in CSF (29/32, 91%, *p* < 0.001). Interestingly, NGS analysis of gDNA from CSF sediment also showed a higher detection rate than FC (18/43, 42%, *p* = 0.03), indicating higher sensitivity in detecting circulating tumor cells (Suppl. Table S4). The redistribution of samples from FC negative to ctDNA/gDNA positive is shown in Fig. 3. Importantly, from 48 FC negative and inconclusive samples, 22 were found positive for CSF-ctDNA and 7 for CSF-gDNA. To account for potential bias due to a subset of cfDNA samples unfeasible for NGS, we conducted the same analysis on 32 CSF samples analyzed simultaneously by both methods. CSF-ctDNA detection remained superior to flow cytometry (91% vs. 25%; *p* < 0.001; Suppl. Table S5 and Suppl. Figure 3). Plasma-ctDNA does not bring such a benefit despite harboring higher levels of cfDNA and ctDNA if present. Of the 15 CSF-ctDNA positive SCNSL samples, only 5 (33%) patients manifested ctDNA in plasma.

### CNSL genotyping by integrative genomic profiling

**Fig. 4:**
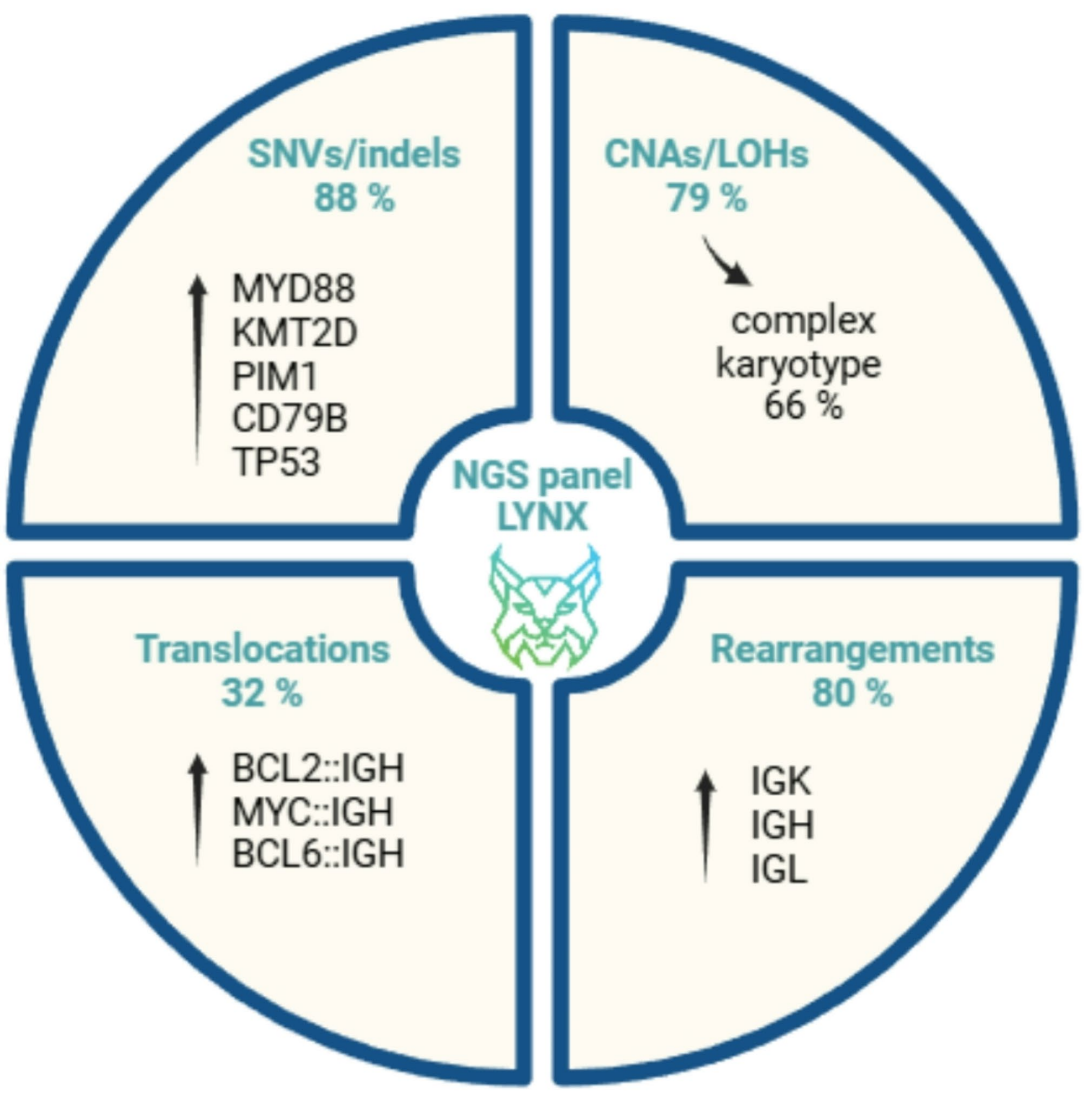
The most frequent findings detected by integrative NGS profiling (LYNX panel) in the CNSL cohort. All proportions (%) were calculated from the entire cohort of 56 patients

In addition to the comprehensive NGS characterization of liquid biopsies, we complemented our tissue-agnostic approach with retrospective FFPE sample analysis to capture a broader scope of aberrations. Extensive genomic heterogeneity was observed irrespective of the material analyzed among CNSL patients. At least one clonal biomarker was found in 91% (51/56) of patients, five did not harbor any somatic alteration included in the NGS panel (Suppl. Table S6). Figure 4 schematically summarizes the findings of our comprehensive CNSL profiling.

The most frequent were mutations in driver genes *MYD88* (*n* = 28), *PIM1* (*n* = 107), *KMT2D* (*n* = 29), *CD79B* (*n* = 22), *TP53* (*n* = 14) found in 50% (28/56), 36% (20/56), 36% (20/56), 25% (14/56), and 18% (10/56) of patients, respectively. Variant allelic frequencies (VAF) varied in both types of liquid biopsies, ranging from fully clonal to subclonal down to the 5% detection limit. The VAF is similar or even higher in CSF than FFPE samples and lower in plasma. In total, we detected 431 mutations; the median number of mutations per patient was 6.0 (range 0–25). 79% (44/56) of patients harbored chromosomal abnormalities, including CNAs and LOHs, and the majority of these (84%, 37/44) had complex karyotypes (≥ 5 defects, illustrative example Suppl. Figure 4). Translocations were observed in one-third of cases (18/56), with the most common being *BCL2*, *MYC*, and *BCL6* rearrangements. Regarding clonality, immunoglobulin (IG) rearrangements were frequently detected (80%, 45/56) in both heavy (IGH) and light (IGK, IGL) chain loci. Moreover, T-cell receptor (TR) rearrangements were also observed (43%, 24/56), albeit being present particularly in lower fractions (< 20% of the respective locus in 21/24 patients), suggesting a reactive expansion of T cells in the tumor microenvironment. A concise overview of detected variants/markers is shown in Suppl. Table S6, NGS detailed results are available upon request.

Looking at the difference in the genetic landscape of PCNSL and SCNSL, only *MYD88* and *PIM1* mutations varied significantly in favor of PCNSL (*p* = 0.007 and *p* = 0.006, respectively). Translocations were found more frequently in SCNSL patients (*p* = 0.024). Chromosomal aberrations were similarly abundant in both CNSL groups. PCNSL uses the VH4-34 gene segment in IGH rearrangements more often than SCNSL (8/25 vs. 3/31, *p* = 0.048).

### *MYD88* L265P variant is enriched in PCNSL and shows a lower CSF-ctDNA detection rate compared to multi-target profiling

Focusing only on *MYD88* hotspot mutation L265P (in canonical transcript ENST00000650905: p.Leu252Pro), we found 26 patients with this variant detected by NGS and/or dPCR in any analyzed material (Suppl. Table S7). CSFs were positive more frequently (16/37 analyzed samples) than plasma, comprising 12/18 (67%) PCNSL and 4/19 (21%) SCNSL (*p* = 0.008). Plasma carried the variant only in four SCNSL cases (4/31) while corresponding CSF was devoid of the variant or not analyzed. Importantly, none of these patients had confirmed CNS disease at the time of investigation, indicating a systemic genetic trait with a potentially higher risk of CNS involvement. The plasma of all PCNSL cases was plain of the variant. By adding the FFPE analysis, we found five extra patients with the L265P mutation (3 PCNSL, 2 SCNSL), where liquid biopsies were not analyzed or were without the variant. In addition to the L265P variant, we also detected non-recurrent *MYD88* mutations in four patients (transcript ENST00000650905: p.Ser206Cys, p.Gln249Ter, p.Gln130Pro, p.Ser230Asn).

*CD79B* variants in any analyzed material were identified in 14 patients (9 PCNSL, 5 SCNSL), mainly in recurrently changed codon for Tyr196. Contrary to the *MYD88* variant, the difference between PCNSL and SCNSL did not reach statistical significance (*p* = 0.123).

Overall, we show that the detection scope of testing a single variant is not as sensitive as covering more alterations in one cfDNA test. Indeed, by using a multi-target NGS approach, the sensitivity for ctDNA presence in CSF reached 90% (26/29), compared to 46% (12/26) in samples where only the *MYD88* variant was analyzed (*p* = 0.001). Enrichment with recurrent *CD79B* variants slightly increased sensitivity to 54% (14/26 = 54%) as some patients harbored concurrent *MYD88* and *CD79B* mutations.

### Clinical relevance

The overall detection rate of tumor infiltration in PCNSL, combining CSF ctDNA and pellet gDNA analysis, was 74% (17/23 samples; Table S2) at diagnosis or relapse, thus contributing to noninvasive diagnostics. Standard methods usually used in parallel (MRI/CT, biopsy, or cytology) confirmed CNS invasion with lymphoma in all cases (Suppl. Table S1). The levels of cfDNA and ctDNA in CSF did not correlate with diagnosis to treatment interval (DTI), which was found to be important in systemic DLBCL [15].

In SCNSL, CNS-IPI was associated with cfDNA level in plasma (*p* < 0.044, *R* = 0.424) but not in CSF. We were not able to correlate ctDNA with CNS-IPI due to a small number of samples. Of the 31 patients enrolled because of proven or suspected CNS involvement, 75% of analyzed samples (18/24) were CSF-ctDNA positive at diagnosis or relapse. Standard methods proved CNS invasion in 45% (14/31) of them, where three of these did not have detectable tumor DNA in CSF. Among 17 cases negative with standard methods, 7 showed CSF-ctDNA and/or CSF-gDNA presence, three were negative and 7 were not eligible for NGS analysis (Suppl. Table S2). When specifically focusing on plasma ctDNA potential, we identified 13 patients with genetic features conferring a higher risk of CNS recurrence, i.e., harboring MCD phenotype, *TP53* mutation, double hit, or *CDKN2A* deletion (del9p) [24, 31, 32]. CNS involvement was proven in five of them (38%) by standard techniques (Suppl. Table S8).

We performed survival analysis in reasonable comparisons based on the above-mentioned results. OS was assessed with regards to the detectable tumor DNA presence in CSF of all CNSL patients and in baseline plasma of SCNSL patients (Suppl. Figure 5A and 5B). In addition, we analyzed time to relapse (EFS) in SCNSL patients in relation to the positivity of tumor DNA in CSF samples from the time of suspected relapse (Suppl. Figure 6). Unfortunately, none of our survival analyses were significant, probably due to cohort disproportion and low sample numbers in compared groups.

## Discussion

The diagnosis of both primary and secondary CNS infiltration by high-grade lymphoma is still hindered by misleading or incomplete results from standard examinations such as tissue biopsy, MRI, and CSF cytology. Here, we present a real-world approach employing routine molecular profiling for noninvasive ctDNA detection in the case of CNS involvement in lymphoma patients. Our aim was to assess the real clinical potential of the diagnostics NGS panel, avoiding the use of laborious ultra-sensitive methods, and also to define potential practical pitfalls.

Previous studies showed the feasibility of ctDNA detection in plasma or CSF but with the limitation of a single-target assay, utilizing clonotypic IG rearrangements or *MYD88* hotspot mutation [12, 20, 22, 23, 25, 33]. The ctDNA detection rate can be improved by multi-target analysis, especially in residual disease monitoring [13, 14, 34]. Our integrative NGS panel, covering the most recurrent lymphoma biomarkers, clearly demonstrated a higher ctDNA detection rate compared to relying solely on *MYD88* and *CD79B* variants. Spatial and tumor heterogeneity, along with ctDNA shedding dynamics, may still impact the detectability of individual variants. Nevertheless, *MYD88* retains high specificity for CNS lymphomas compared to other brain tumors and infectious or demyelinating diseases [12, 13, 35]. In addition, analyzing ctDNA eliminates the need for an initial tumor biopsy (i.e., tumor-agnostic approach) to infer patient-specific aberrations and targets for possible follow-up monitoring, as demonstrated in our study and by Heger et al. [14]. The spectrum of genomic lesions found in our cohort was typical for lymphoma-invading CNS [31, 36–40], with a remarkably high frequency of complex karyotypes. PCNSL and SCNSL were expected to be very similar as both need to adapt to the CNS environment; only *MYD88* and *PIM1* mutations and lymphoma translocations showed differing abundances. Notably, the predominant use of the VH4-34 gene segment in clonal IG rearrangement found in the PCNSL group supports the hypothesis of chronic BCR stimulation by a specific antigen in immune-privileged sites [7, 41]. Frequently observed TCR rearrangements correspond with a typical infiltration of CNSL microenvironment with cytotoxic T cells [42].

While plasma is a reliable material for cfDNA analysis in systemic DLBCL, its use is debatable in CNSL and depends on the intended application and method sensitivity. CSF seems more appropriate due to localization in the proximity of the tumor and the blood-brain barrier preventing ctDNA from spreading into plasma. Indeed, several studies showed CSF superiority over plasma in ctDNA detection rate [12, 20, 21]. However, two recent papers provided opposing results using ultrasensitive methods [13, 14]. Our routine testing emphasized the relevance of CSF use, demonstrating a significantly higher ctDNA detection rate over plasma at the time of CNS infiltration by lymphoma. Intriguingly, in SCNSL, half of the patients with reliably detected ctDNA in CSF had no CNS involvement confirmed by standard methods. These cases can represent subclinical lymphoma infiltration, which deserves careful patient monitoring. In addition, we provide pilot data for plasma ctDNA potential in revealing DLBCL patients with a higher risk of CNS recurrence that was shown previously only on tissue samples [24, 31, 32]. The presence of CNS-tropic ctDNA aberrations in plasma was associated with a 38% risk of CNS recurrence in our cohort, similar to 29% in Olszewski et al. [20] analyzing clonotypic ctDNA. Molecular characteristics like ABC cell-of-origin, MCD phenotype, *TP53* mutation, double hit, or *CDKN2A* deletion (del9p) combined with clinical parameters could help to stratify patients and rationalize the use of CNS prophylaxis.

Current laboratory practice relies on cell-based methods to prove CNS involvement in lymphoma. However, tumor cell count can be subtle in CSF, reflecting the low sensitivity of conventional cytology and flow cytometry results demonstrated in our study and others [12, 13, 28]. In line with that, we observed a higher detection rate from liquid fraction (cfDNA) than from cell pellet (gDNA) of CSF. The parallel analysis of both compartments or pooled DNA can improve diagnostic yield [13, 20, 28], although with the awareness of increased time and expenses for sample processing. Like with CTCs, the CSF volume represents a limitation to overall test sensitivity in terms of ctDNA content [43]. In our study, a threshold for NGS CSF-cfDNA analysis is 25 ng with 90% sensitivity of ctDNA detection, comparable to Mutter et al. [13] with 33 ng to reach 100% detection with ultrasensitive targeted sequencing. Our CSF-ctDNA detection rate could be increased with more eligible samples, as in half of all cases, 10 ml of CSF was not enough to yield sufficient cfDNA amount. The guidelines for neurological examinations allow for up to 30 ml of CSF to be collected [44] without increasing the risk of complications, which would highly probably augment the diagnostic yield. Thus, for clinical settings, the sample volume is crucial, and the analytical method must balance sensitivity, specificity, and practical considerations such as cost and turnaround time.

The clinical benefit of cfDNA analysis in PCNSL resides indisputably in the possibility of diagnosing the lymphoma without risky brain biopsy. We demonstrated a satisfactory CSF-ctDNA detection rate where three-quarters of patients could be diagnosed non-invasively by affordable routine NGS method, with the advantage of combining genotyping and clonality assessment in one test. Notably, the use of corticosteroids before biopsy is still controversial regarding diagnostic yield and delay [11, 45], but we did not observe any association with the subsequent ctDNA levels. In SCNSL, the CSF-ctDNA detection level was higher (75%) compared to standard imaging and laboratory techniques (45%), promising a complementary method that can foresee patients with possible CNS involvement not yet revealed by routine methods. We are aware that clinical outcome associations need to be performed in a more extensive and uniform cohort, ideally enrolling balanced CNS-IPI groups, as patients with low and intermediate risk can also experience CNS relapse [32, 46].

Given the shortcomings of our study, we propose several optimization strategies to enhance the clinical utility of cfDNA analysis in CNS lymphoma (summarized in Table 2). The exclusion of approximately one-third of CSF samples due to insufficient cfDNA may have affected study accuracy, emphasizing the need to increase CSF collection volume to at least 20 mL to improve the overall sensitivity. While plasma is a readily accessible biofluid, our findings confirm that it is not a reliable material for routine CNS lymphoma diagnostics. CSF remains the preferred source for ctDNA analysis, offering higher accuracy while still being considerably less invasive than a tissue biopsy. Additionally, longitudinal CSF-ctDNA monitoring using ultra-sensitive methodologies in prospective studies could improve early detection of CNS relapse, as supported by previous research [12, 13, 20]. Lastly, despite our three-year prospective collection, the cohort size was insufficient to establish definitive correlations between ctDNA levels and clinical outcomes. Given the rarity of CNS lymphoma, larger multi-center studies will be essential to validate the prognostic and predictive value of cfDNA analysis. Although prospective cfDNA clinical trials are ongoing [47], standardization challenges remain [48, 49], and solid molecular endpoints still need to be defined. Overcoming these hurdles will be key to integrating cfDNA-based diagnostics into routine clinical practice and maximizing their impact on patient care.

**Table 2:** Study weaknesses and strategies for clinical implementation of cfDNA analysis in CNS lymphoma

Shortcoming	Optimization strategy
High rate of CSF samples with insufficient cfDNA level (≈ 30%)	Increase CSF collection volume to at least 20 mL to enhance the success rate of ctDNA analysis and overall sensitivity.
Restricted plasma utility for CNS lymphoma diagnostics	Prioritize CSF as the preferred source for ctDNA analysis in routine setting, providing higher diagnostic accuracy.
Lack of longitudinal sampling for CSF-ctDNA monitoring	Implement ultra-sensitive methodologies in prospective studies to improve early CNS relapse detection and assess clinical utility.
Small cohort size limiting correlation with clinical outcomes	Conduct larger multi-center studies to validate the prognostic and predictive value of cfDNA analysis in CNS lymphoma.

## Conclusions

The present study demonstrates the real-world importance of ctDNA as a useful biomarker, refining and improving CNSL diagnostics. Our multi-target NGS profiling offers a substantial ctDNA detection yield in a routine and affordable way, highlighting CSF superiority over plasma to determine CNS involvement in aggressive lymphomas. The tumor-agnostic cfDNA analysis is feasible and outperforms conventional laboratory techniques in terms of sensitivity while keeping the procedure minimally invasive. For clinical implementation, the CSF sample volume and properly selected analytical method, which must equalize accuracy and applicability, are crucial in effectively implementing the cfDNA approach in routine CNSL patient management. We envision that ctDNA profiling, in combination with MRI imaging and clinical criteria, could effectively identify patients with a high probability of current or possible future CNS infiltration.

## Electronic supplementary material

Below is the link to the electronic supplementary material.

Supplementary Material 1

Supplementary Material 2

## Data Availability

The original contributions presented in the study are included in the article. The detailed datasets obtained during the study are available from the corresponding author upon reasonable request.

## Abbreviations

cfDNA: Cell-free DNA
CSF: Cerebrospinal fluid
CK: Complex karyotype
CNA: Copy number abnormality
cnLOH: Copy-neutral loss of heterozygosity
CNS: Central nervous system
CNSL: Central nervous system lymphoma
CT: Computed tomography
CTC: Circulating tumor cell
ctDNA: Circulating tumor DNA
DLBCL: Diffuse large B-cell lymphoma
dPCR: Digital PCR
EFS: Event-free survival
FC: Flow cytometry
FFPE: Formalin-fixed paraffin-embedded
gDNA: Genomic DNA
hGE: Haploid genome equivalent
IG: Immunoglobulin
LYNX: LYmphoid NeXt-Generation Sequencing
MRI: Magnetic resonance imaging
NGS: Next-generation sequencing
OS: Overall survival
PCNSL: Primary CNS lymphoma
PET: Positron emission tomography
SCNSL: Secondary CNS lymphoma
TR: T-cell receptor
