# Supplementary material for: Reliable detection of CNS lymphoma-derived circulating tumor DNA in cerebrospinal fluid using multi-biomarker NGS profiling: insights from a real-world study
Source: Biomark Res. 2025 May 9;13:71. doi: 10.1186/s40364-025-00777-z (PMC12065147; doi:10.1186/s40364-025-00777-z)

Supplementary Figures

Suppl. Figure 1: Target regions of the updated and validated version of the LYNX panel.

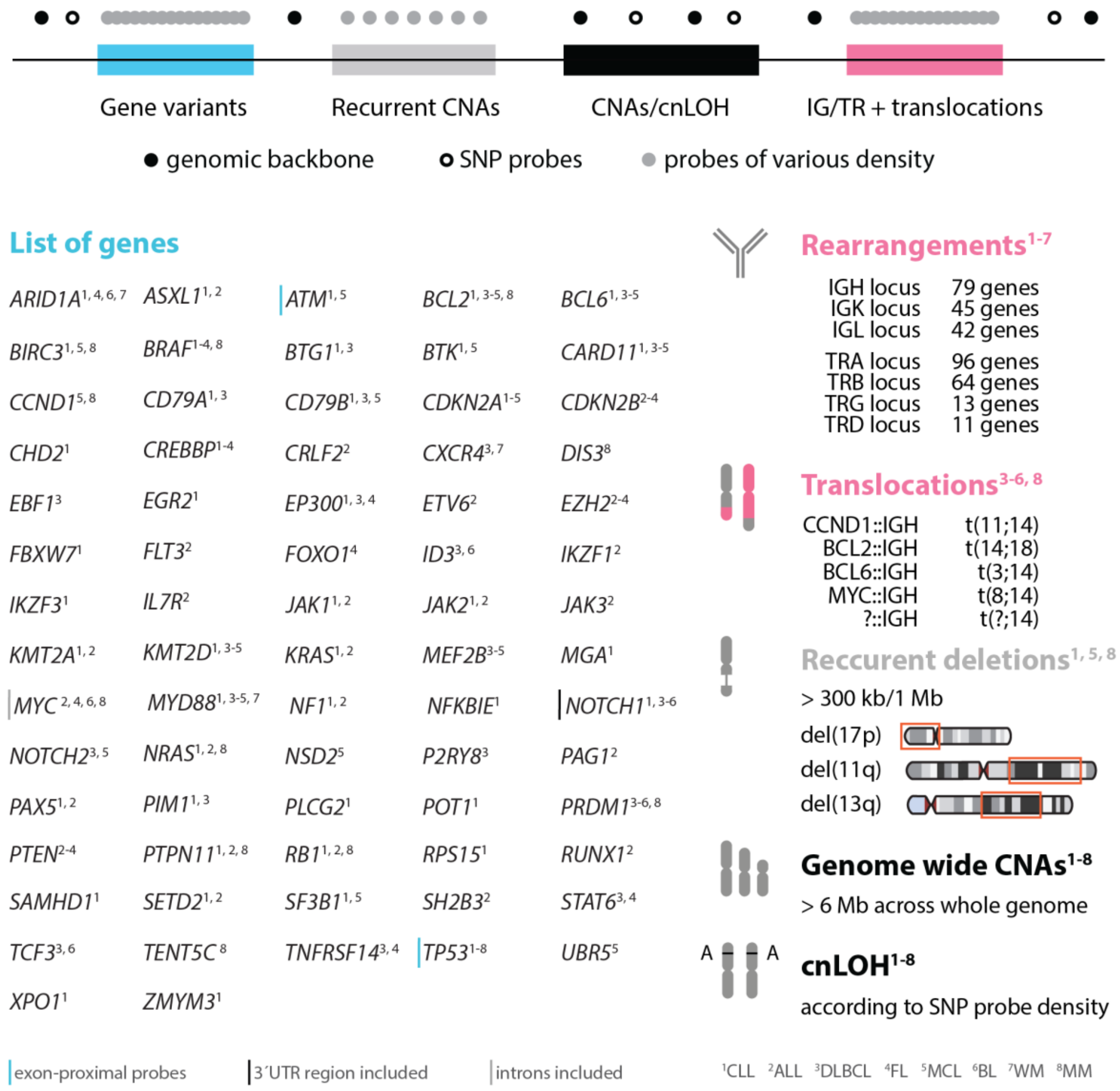

Suppl. Figure 2: Differences in the level of cfDNA and ctDNA between PCNSL and SCNSL groups. A) total cfDNA amount (ng) in CSF (p=0.99), B) total cfDNA amount (ng) in plasma (p=0.08), and C) ctDNA fraction (log hGE/ml) in CSF (p=0.68). Medians (red lines) are shown; * outliers.

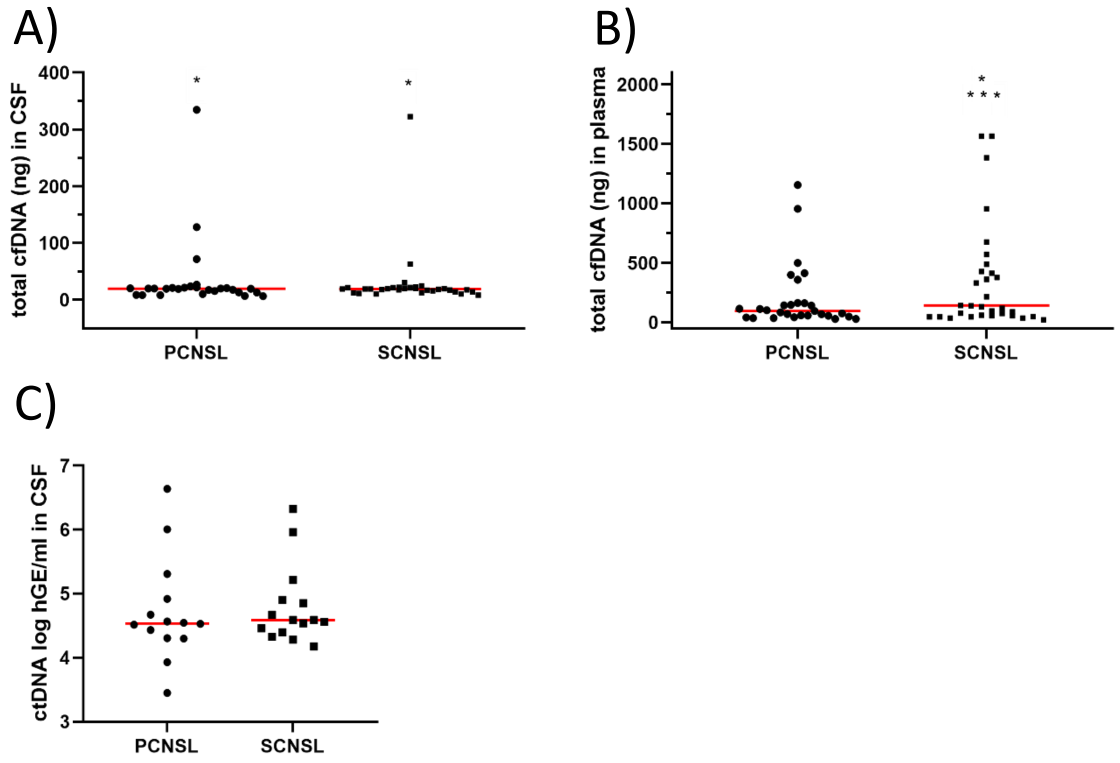

Suppl. Figure 3: Comparison of the sensitivity to detect tumor infiltration by flow cytometry and CSF-ctDNA analysis restricted to patients who underwent both methods. na* not analyzed due to the low amount of gDNA from CSF pellet for NGS.

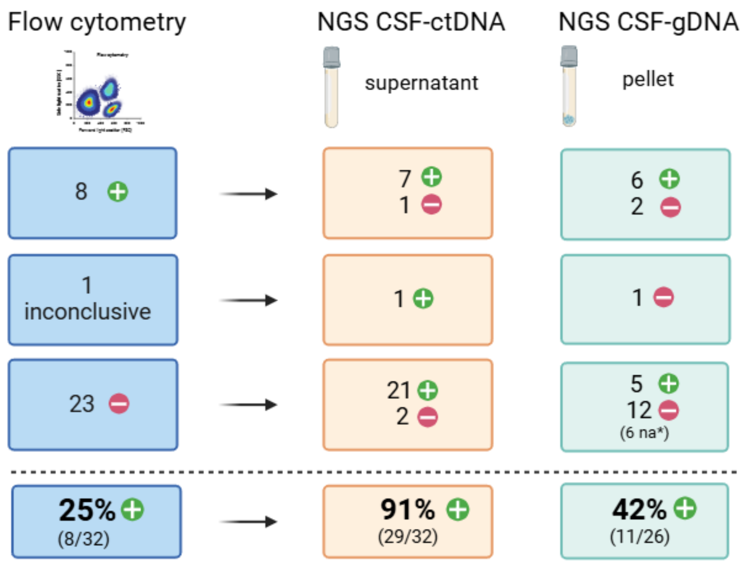

Suppl. Figure 4: An illustrative example of complex karyotype (≥ 5 defects) detected in CSF (A) and FFPE (B) samples of patient no. 113.

A)

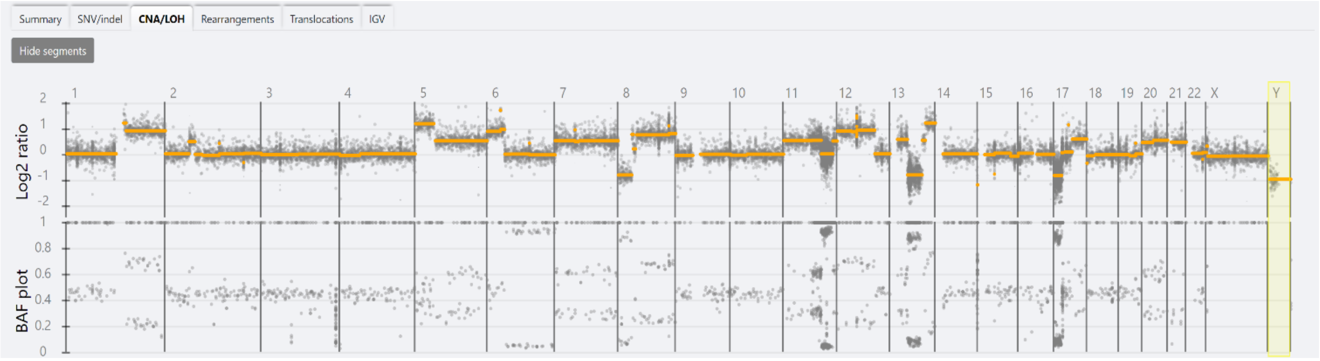

B)

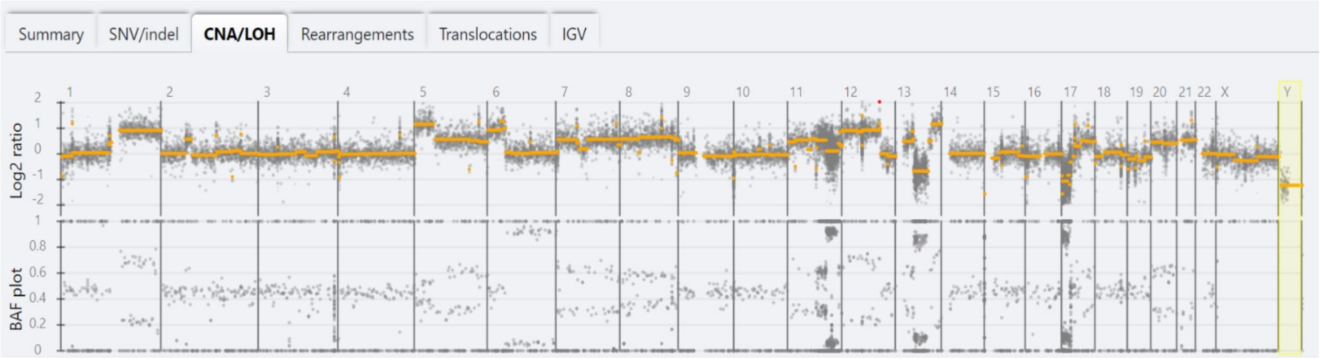

Suppl. Figure 5: Overall survival according to detectable tumor DNA in A) CSF ctDNA/gDNA of all CNSL patients and B) baseline plasma ctDNA of SCNSL patients. Both analyses were not significant.

A)

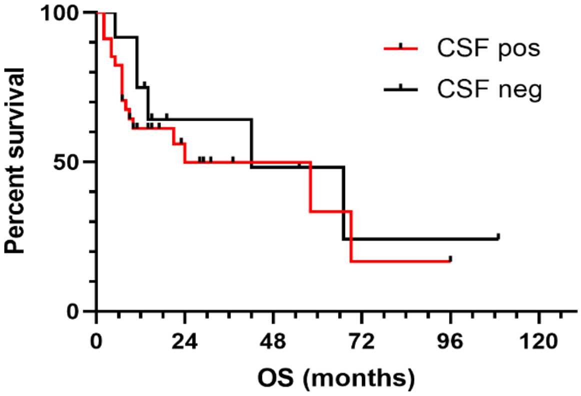

B)

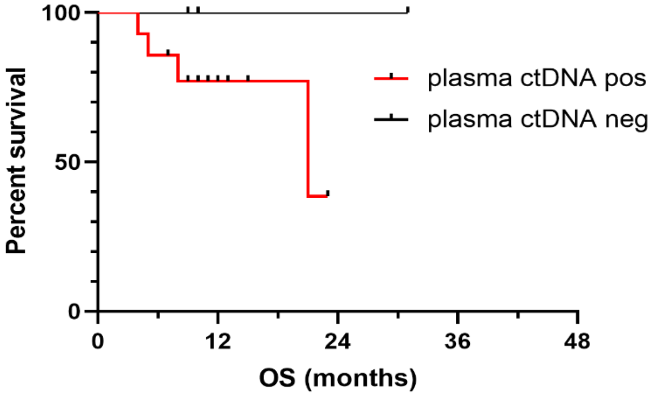

Suppl. Figure 6: Event-free survival (EFS) in SCNSL patients experiencing CNS relapse according to the presence of detectable tumor DNA (ctDNA/gDNA) in CSF. Median 15 vs 40 months, non-significant.

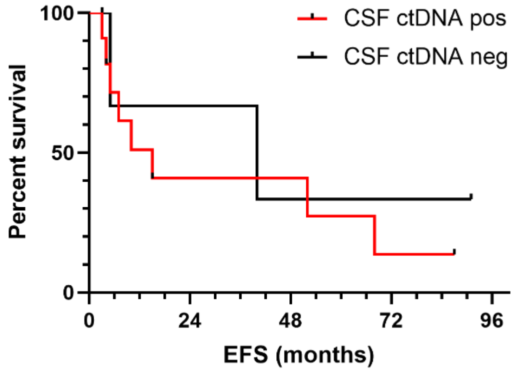

Supplement: Supplementary file 2 — Supplementary Material 2 [file 40364_2025_777_MOESM2_ESM.pdf]
